# Institutionalize Reciprocity to Overcome the Public Goods Provision Problem

**DOI:** 10.1371/journal.pone.0154321

**Published:** 2016-06-01

**Authors:** Hiroki Ozono, Yoshio Kamijo, Kazumi Shimizu

**Affiliations:** 1 Faculty of Law, Economics and Humanities, Kagoshima University, Kagoshima, Japan; 2 School of Economics and Management, Kochi University of Technology, Kochi, Japan; 3 School of Political Science and Economics, Waseda University, Tokyo, Japan; Chinese Academy of Science, CHINA

## Abstract

Cooperation is fundamental to human societies, and one of the important paths for its emergence and maintenance is reciprocity. In prisoner’s dilemma (PD) experiments, reciprocal strategies are often effective at attaining and maintaining high cooperation. In many public goods (PG) games or *n*-person PD experiments, however, reciprocal strategies are not successful at engendering cooperation. In the present paper, we attribute this difficulty to a coordination problem against free riding among reciprocators: Because it is difficult for the reciprocators to coordinate their behaviors against free riders, this may lead to inequality among players, which will demotivate them from cooperating in future rounds. We propose a new mechanism, institutionalized reciprocity (IR), which refers to embedding the reciprocal strategy as an institution (i.e., institutionalizing the reciprocal strategy). We experimentally demonstrate that IR can prevent groups of reciprocators from falling into coordination failure and achieve high cooperation in PG games. In conclusion, we argue that a natural extension of the present study will be to investigate the possibility of IR to serve as a collective punishment system.

## Introduction

Cooperation is fundamental to human societies, and one of the important paths for its emergence and maintenance is reciprocity: responding to kindness with kindness and to unkindness with unkindness (e.g., [1–3]). Regarding the finitely repeated two-person prisoner’s dilemma (hereafter PD), standard game theory states that the unique subgame perfect outcome is defection in every game. Experimental and field evidence, however, contest this prediction. In PD experiments, reciprocal strategies such as tit for tat (TFT) ([4]) and raise the stakes (RTS) ([5]) are often effective at attaining high cooperation (e.g., [6–10]).

In the RTS strategy, a player raises the stake (contribution) when mutual cooperation is achieved in the previous period and reduces it to a level equal to the other player’s contribution in the previous period when the other player’s contribution is lower than his or hers in the previous period. Since the 1980s, finitely repeated game theories have shown how a finite number of repetitions might allow cooperation (e.g., [11,12]). They show that if there are sufficient beliefs among players that “reciprocal” type strategies, like “tit-for-tat” or “trigger” exist, then cooperation can be established early in the game by rational and selfish decision making.

Contrary to the success in PD experiments (where the number of participants is limited to two), many public goods (hereafter, PG) games or *n*-person PD experiments (where the number of participants exceeds two) have clearly revealed the difficulty in maintaining a high level of cooperation (e.g., [13–17]). Grujić et al. [15] directly compare the differences between the outcomes of PD experiments with two to three, four, and five persons and reveal that two-person PD games were more likely to reach high cooperation, unlike those with more than two persons. Why does reciprocal strategy not work in the same way in PD experiments with more than two persons or in PG games as it does in two-person PD games? We are of the view that this puzzle needs solving, because from the standard game theoretical view, both games are structurally identical (while the dominant strategy is non-cooperation, cooperation by all players leads to the most efficient situation in social terms) and because experimental and field evidence have shown that a considerable percentage of people were categorized as some type of reciprocator even in PD games with more than two people and in PG games (e.g., [15, 18–21]). For example, Fischbacher et al.’ study [20] reported that 51% of their participants were conditional cooperators, a type of reciprocator.

The literature regarding the effect of group size on cooperation in a PG game explains the negative effects of group size on cooperation as follows. Olson’s study [22] states that the factors of social pressure and social incentives can be more conducive to establishing cooperation in smaller than in larger groups. Kim and Walker discuss that individuals in small groups have greater recognition that their free riding may have a negative impact on others’ willingness to cooperate, and thus, they are more likely to refrain from free riding ([23]). de Oliveira et al.’s study [24] suggests that if a group contains one non-cooperator (a “bad apple”), cooperation will unravel, because many individuals are willing to cooperate only as long as others do so as well. Further, the probability of the existence of a “bad apple” becomes higher as group size increases. Grujić et al. [15] argue that in PD games with more than two people, defection as a form of reciprocity hurts not only partners who defected in the previous period but also partners who cooperated; thus, reciprocity does not work well. For a literature review on the group size effect, see Nosenzo et al.’s [25].

In the present paper, we develop the idea of Grujić et al. [15], and we argue that there is a coordination problem against free riding among reciprocators and that coordination failure causes cooperation failure in a sizable group. Moreover, we propose and experimentally demonstrate that a new mechanism, institutionalized reciprocity (IR), can prevent reciprocators in groups from falling into coordination failure and achieve high cooperation in a PG game.

## Coordination Failure and Institutionalized Reciprocity

Imagine that in a repeated two-person PD with multiple choices, Player 1 free rode and made a lower contribution than Player 2 in some round, and in the subsequent round, Player 2, a reciprocator, also chose to lower her cooperation level to punish Player 1. If the initial free rider considers the low contribution of Player 2 as punishment, she may increase her cooperation level in future rounds, and Player 2 will reciprocate her cooperation. In other words, in two-person repeated PDs, the likelihood of maintaining high cooperation depends on whether the free rider understands the intention of the reciprocator. In cases of games with more than two persons, however, the situation changes drastically.

Suppose that in a repeated three-person PG game with multiple choices from 0 to 10, there are two reciprocators, including you, and one defector. In the first period, you and another reciprocator contributed 10, and the other, 0. As a reciprocator, how much would you contribute in the next period? There are several options: contributing 10 to reciprocate the other full contributor, 0 to reciprocate the free rider, or around 5 (= 0/2 + 10/2), as an average of the other two players’ contributions. If coordination fails among reciprocators, such that you choose 5 but the other chooses 0, in the next period, you lose your benefit, because her low cooperation, even if it is aimed to punish the free rider, will hurt the other non-free riding player equally, that is you. As coordination failure among reciprocators causes inequality among reciprocators, the inequality will demotivate the “punished” reciprocator from cooperating in future rounds. Many studies have shown that humans tend to be inequality aversive (e.g., [26–28]). Some researchers show that participants in PG experiments reduce their contribution levels because they are averse to inequality ([29, 30]).

As mentioned above, in a two-person repeated PD, it is important for the free rider to adequately understand the reciprocator’s intention to realize and maintain high cooperation. In addition, in a three-person repeated PD, it is important for reciprocators to understand the other players’ intentions, to coordinate their reciprocal behavior against the free rider and attain a high level of cooperation. This difficulty in achieving coordination among reciprocators is amplified as the number of players increases, as reported from observations of many coordination game experiments ([31]). The multiplied difficulty in coordination will lead players to lose the perspective of mutual cooperation in the long term. Some studies have shown that uncertainties about others’ intentions or strategies reduce cooperative behavior. For example, de Oliveira et al. [24] reveal that when all participants know that all members are reciprocators, they cooperate more than when they do not know. In this manner, even if the game includes reciprocators, they will be more likely to lose the incentive to cooperate in a group size exceeding two players. This problem will also drive rational free riders to continue non-cooperation because they cannot foresee cooperation being more profitable than free riding in the long term.

We propose institutionalized reciprocity to overcome the problem of coordination failure among reciprocators and to enhance cooperation among reciprocators. If a unique reciprocal strategy is institutionalized, various reciprocal behaviors of the players can converge, and reciprocators can share a belief about how much they should reciprocate and then coordinate their behavior against free riders. This coordinated reciprocal behavior will also effectively lead rational free riders to change their free riding into cooperation, to maximize their long-term profit. Let us explain IR more concretely.

In our study, participants play a four-person repeated multiple-choice PG game, and each player simultaneously chooses her contribution from {0, 10,…, 100}. We choose a four-person version of the PG game, not only because this group size exceeds two, but also because we may then compare our results with those of previous PG game experiments (see [32]). We apply the RTS strategy as a reciprocal rule: In the first period, the upper bound is 10, and players can decide whether to contribute 0 or 10. When all members contribute to the upper bound, the upper bound of the next period will rise gradually by 10 until it reaches 100. When even one player fails to meet the upper bound, the bound in the next period will be reduced to a level equal to the minimum contribution in the previous period. The reason we choose the RTS strategy as a unique reciprocal strategy is mainly because this strategy theoretically and empirically leads to high cooperation in multiple-choice two-person PD games ([5, 10]). In addition, Kamijo et al.’s study [33] reveals a mechanism called the embedded RTS strategy, which could overcome the coordination failure in minimum effort coordination games. A potential motivation of the present study is to investigate whether the mechanism proposed by [33] can work in PG games as well as coordination games.

Internalizing the RTS strategy in society will force society members to use a unique reciprocal strategy. Hence, it can guide reciprocators on how to behave against free riders and encourage a rational player to be cooperative so as to maximize her long-term profit.

## Experimental Conditions—Two Types of Institutionalized Reciprocity

In this paper, we describe experiments of a four-person repeated multiple-choice PG game, to investigate whether IR could solve the PG problem. In this game, each player simultaneously chooses her contribution from {0, 10, …, 100}, which will be extracted from her endowment of 100 points. The contributions are multiplied by 1.6 and split evenly among the four group members.

We design the following conditions as IR conditions: Standard IR condition (hereafter, S-IR) and no limit IR condition (hereafter, NL-IR). The difference between the two IR conditions is that while a player cannot contribute more than the target (an upper bound) in S-IR, players can choose a contribution higher than the target in NL-IR. In both NL-IR and S-IR, the target contribution changes according to the RTS strategy. Thus, in the first period, the target contribution was set as 10 points. After the second period, the target contribution was increased by 10 points provided that all the players exceeded or met the target contribution in the previous period. Otherwise, the target was adjusted to the minimum contribution among all the group players in the previous period. Note that while the target contribution is just a nominal target in NL-IR, it is a substantive boundary in S-IR.

As the control condition (hereafter, CON), we prepare a typical repeated PG game, where players can choose their contribution levels between 0 and 100 without a strict constraint. In CON and NL-IR, all the players can choose from 0 to 100 points in every period, while players in S-IR are not allowed to exceed the target contribution. In fact, under S-IR, NL-IR, and CON, if players participating in a repeated public goods game have a self-regarding preference that is common knowledge, the standard game-theoretic prediction is that they free ride in every stage of the game, which can be proven by backward induction.

Although our main purpose is to verify if the PG game with IR will achieve higher cooperation than the typical PG game (CON), why did we design NL-IR? We did so because we are of the opinion that the restriction of maximum contribution must play an important role in institutionalizing reciprocity: All the participants can believe that reciprocators’ contributions will converge to a certain level. Unlike S-IR, if they want, the participants in NL-IR can contribute beyond the announced target contribution from the beginning of the game, since the upper bound is not compelling in NL-IR. Thus, a free rider would be more likely to emerge in NL-IR as early as the initial period because low cooperation as a punishment against free riding would be not effective; the target contribution being adjusted to the minimum contribution in the previous period would be in name only, and it cannot pose a threat of a loss in future benefit. Even if there are no free riders in the first period, free riding behavior will occur earlier in NL-IR than in S-IR because contribution levels might vary among participants in NL-IR, and there is larger room for rational free riders to exploit cooperative members. There is a practical reason as well for the inclusion of NL-IR in our design. As one of the purposes of the present study is to propose a feasible mechanism that enables people to cooperate in PG games, it is worth examining whether IR can work under a more general condition, where players can contribute without constraint in each period.

## Method

The Waseda University Ethical Review Board specifically approved this study.

### Treatments

We conducted three types of PG game experiments: S-IR, NL-IR, and CON. In every treatment, the PG game was repeated 20 times; thus, each treatment was spread over 20 periods, and there were 168 participants in total.

Each participant was assigned to one of the three conditions. Of the total, 60 participated in S-IR, 64 in NL-IR, and 44 in CON. Participants were separated into groups of four, and group members were fixed throughout the duration of the experiment according to a partner matching design. Thus, there were 15 independent groups for S-IR, 16 for NL-IR, and 11 for CON.

### Participants

We recruited 168 (= 60 + 64 + 44) undergraduate students from various disciplines. All participants were recruited from Waseda University via its portal site. Written informed consent was obtained from all participants. We conducted the experiment, composed of 11 sessions, from November 2012 to January 2013.

### Procedures

In all treatments, participants were randomly assigned to laboratory booths at the beginning of the experiment. These booths separated participants in order to ensure that every individual made her decision anonymously and independently. Participants were provided with written instructions explaining the game, payoffs, and procedures. In particular, we explained that the targets for contributions would vary across periods. The instructions used neutral wording, as is common practice in experimental economics. After reading the instructions, participants were tested to confirm that they understood the rules and knew how to calculate their payoffs. We did not start the experiment until all participants had answered all questions correctly. Therefore, all participants completely understood the rules of all transactions and were able to calculate their payoffs.

Participants were then randomly and anonymously allocated to groups of four, and these groups played the PG game. Group composition remained unchanged throughout the 20 periods in order to retain statistically independent groups. Each group member had to simultaneously determine her contribution level on the computer screen. After their decisions were made, feedback was provided to participants, such as their current payoffs and the contributions made by each member of the group in that period. After each experiment, all participants answered a demographic questionnaire.

We used z-Tree software ([34]) to conduct the experiments. Each session took approximately 1 hour to complete on average. Participants’ earnings were the sum of points gained over all the 20 periods, exchanged at a rate of 10 points = 5 yen. Participants were also paid a participation fee of 500 yen. The mean payment per participant was 1276 yen (12.76, evaluated at 1 US dollar = 100 yen). The maximum was 1784 yen (17.84 US dollars), and the minimum, 781 yen (7.8 US dollars).

### Hypothesis development

The following hypotheses about cooperation success and failure in our three experimental treatments are based on the explanation in “Experimental Conditions.”

#### Hypothesis 1

For period *t* ≥ 10, the contribution level per group is greater for S-IR compared to (a) CON and (b) NL-IR (after the 10^th^ period, the theoretical maximum contribution was identical across the three treatments, at 100 points).

#### Hypothesis 2

The total profit per group is greater for S-IR compared to (a) CON and (b) NL-IR.

## Results

The data are analyzed at the group level to take into account interdependence of outcomes for members of a given group, excluding cases when we need not be wary of interdependence. All the multiple comparison results of the non-parametric analyses are corrected by the Bonferroni method.

### Analysis of contribution level and total profit

**Fig 1:**
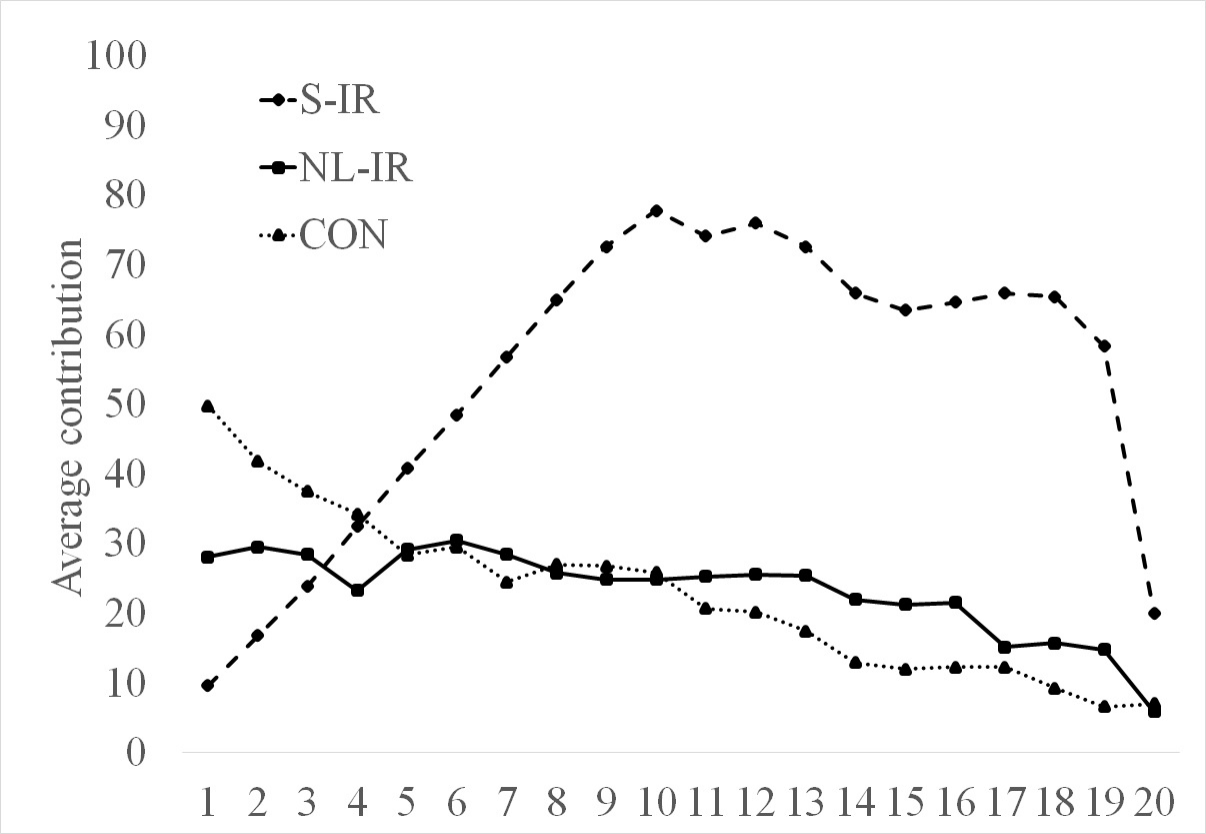
Comparison of average contributions for all treatments in periods 1–20. Note: We use group-level data.

First, the transition of participant contribution levels in each treatment is reported in Fig 1. We see that in S-IR, except for the final period, the contribution increased for the first 10 periods and continued to remain at a higher level, whereas in NL-IR and CON, the contribution level did not increase or decreased weakly across periods (the participants in our experiments knew the game would end in the 20^th^ period, thus causing the end effect to occur).

Table 1 shows the results of the exact rank–sum test, comparing the average of participants’ contributions at the group level. In the first part of the game (*t* ≤ 9), the contributions in S-IR, NL-IR, and CON are not significantly different. In contrast, in the second part of the game (10 ≤ *t* ≤ 19), the contribution levels appear in the order S-IR > NL-IR ≈ CON (CON versus NL-IR, n.s.; CON versus S-IR, *p* value = 0.000; NL-IR versus S-IR, *p* value = 0.001). These results are consistent with Hypotheses 1(a) and 1(b), which imply that S-IR, but not NL-IR and CON, has the power to uphold cooperation in PG games.

**Table 1:** Results of the rank–sum test comparing group efforts.

	The first part of the game (*t* ≤ 9)			The second part of the game (10 ≤ *t* ≤ 19)		
	CON	NL-IR	S-IR	CON	NL-IR	S-IR
CON		102.5(n.s.)	56(n.s)		56(n.s)	17***(.0009)
NL-IR			76(n.s.)			22***(.003)

Note: The numbers show *W* statistics. *p* values are given in parentheses.

*, **, and *** indicate significance at the 10, 5, and 1% levels, respectively.

As for Hypothesis 2, we examine the difference in total profits among conditions. The average total profit for S-IR, NL-IR, and CON is 2642.8, 2279.6, and 2273.9, respectively. Table 2 shows the results of the exact rank–sum test at the group level. It reveals that the total profit for S-IR is greater than that for NL-IR and CON (*p* values < 0.001). Thus, we confirm Hypotheses 2(a) (S-IR > CON) and 2(b) (S-IR > NL-IR) regarding the total profits of the groups.

**Table 2:** Results of the rank–sum test comparing group profits throughout 20 periods.

	CON	NL-IR	S-IR
CON		88.5(n.s)	138***(.008)
NL-IR			197***(.005)

Note: The numbers show *W* statistics. *p* values are given in parentheses.

*, **, and *** indicate significance at the 10, 5, and 1% levels, respectively.

### Comparison between S-IR and NL-IR

We investigate the free riding behavior in the first period. The percentage of zero contributors in the first period was 3.3% (2/60) in S-IR and as high as 15.6% (10/64) in NL-IR, indicating a significant difference (Fisher’s exact test: *p* = 0.031). In addition, it is worth pointing out that 45.3% (29/64) participants in NL-IR contributed above the target contribution (10 points at the first period), which means that many participants in NL-IR ignored the target contribution from the beginning.

We analyze the later periods as well. We find that 13 of 15 groups for S-IR and 7 of 16 groups for NL-IR did not have zero contributors in the first period. In these groups, the first appearance of a free rider, a player who contributed less than the target contribution for the first time in the group, occurred at periods 2, 2, 8, 11, 12, 13, 15, 18, 19, 20, 20, 20, and 20 in S-IR and at periods 3, 3, 3, 5, 6, 8, and 12 in NL-IR. The exact rank–sum test thus shows that the first appearance of a free rider occurs significantly later in S-IR than in NL-IR (*p* value = 0.024).

## Conclusion and Discussion

We conclude the paper by summarizing our findings and discussing topics related to IR in the following subsections.

### Institutionalized reciprocity can solve a public goods provision problem

Stimulated by the idea of Grujić et al.’s paper [15], we attribute the main cause of cooperation failure in a sizable group to the coordination problem among reciprocators. We are of the view that the IR mechanism can overcome this problem because a unique reciprocal strategy embedded as an institution can converge reciprocal behaviors of various players, especially in terms of how they should coordinate their behavior against free riders. As our experimental results show, S-IR outperforms CON and NL-IR.

As predicted, the data show that free riders appeared earlier and more frequently in NL-IR than S-IR, and thus, high cooperation was not achieved; as the target contribution is merely nominal in NL-IR, players are allowed to contribute more than the target indicates. As the announced target is not compelling in NL-IR, it is difficult for the other players to coordinate punishment against free riders. Further, rational players may have more incentive to exploit cooperators, as they can expect that some players will contribute more than the indicated target (in the first period, 45.3% of players contributed more than 10 points in NL-IR). Considering that the unique difference between these two conditions is the credibility of the announced target, we can attribute their contrasting performance to this credibility, based on which players can construct a shared belief about their strategies.

### Restricting contributions to the target

To be fair, although we think that the IR mechanism can garner good performance/cooperation, we should note that it worked under the condition in which players could not contribute more than the target. This suggests that the IR mechanism needs to restrain participants from excessive cooperation, that is, from contributing more than the target. In some cases, we can easily suppose this sort of restraint. For example, tax, pension, or fees for public services should be paid to the extent they are determined institutionally. Here, the problem of excessive cooperation is avoided in advance. When administrative restraint does not exist, the following three candidate ideas may be considered to achieve the necessary restraint.

The first idea concerns punishment to excessive cooperators, those who contribute more than the target. The punishment will certainly raise another question: Who will pay for the cost of the punishment? However, it is worth pointing out that the punishment is directed only toward excessive cooperators and not toward free riders. In reality, excessive cooperators are much fewer in number than free riders in the public goods provision case because excessive cooperation in itself is costly. Therefore, the actual cost of punishment to excessive cooperators should be quite low. Thus, this cost might not pose a serious problem. Punishment to excessive cooperators, namely those who behave prosocially, can be considered as “antisocial punishment.” While previous studies showed that antisocial punishment harms cooperation (e.g., [35]), our idea suggests that antisocial punishment may help achieve cooperation in a specific situation like IR.

The second idea entails a leader or authoritative figure making announcements about or recommendations for target contributions. The effect of a leader (an authority) is a popular topic in psychology; a reputed study in social psychology reveals that people tend to obey a leader or an authority even if her command is seemingly cruel ([36]). Leadership studies in economics show that under voluntary contribution settings, a non-binding suggestion about contributions by a leader can influence a member’s cooperation rate. For example, Levy et al. [37] report how a leader makes an initial non-binding announcement to the group about the amount to be contributed, and although weak, this form of leadership exerts a positive impact on members’ cooperation. Based on their study, we conclude that players, including excessive cooperators, are likely to follow their leader’s announcement regarding target contributions, thus ensuring that the IR mechanism will work.

The third idea concerns communication among group members. Communication facilitates sharing expectations in a group (e.g., [38]). If members can communicate with each other and share the belief that all the other members will honor the target contribution, excessive cooperation might be avoided because members would understand that excessive cooperation is not more profitable than obedient cooperation, that is, contributing to the target. Solution by communication, however, would be applicable to a small group, within which the members can communicate successfully with each other. Although we think these candidate ideas are feasible and will produce the expected effect, whether they can restrain excessive cooperation and uphold high-level cooperation in a PG game needs to be examined.

### IR as a collective punishment system

A natural extension of the present study would be to investigate collective punishment regimes. In the following paragraphs, we briefly discuss the possible advantages and disadvantages of IR as a collective punishment system.

In IR, as the current cooperation failure lowers the bound in the next period, it results in a loss of the future benefit that could have been obtained through successful cooperation. It means that when an individual violates a rule (contribution at a targeted level), not just the individual but other members of that person’s group as well are collectively punished by IR. Thus, IR can be considered a collective punishment system. Despite our efforts, we were unable to locate experimental studies in this area. We did, however, review theoretical/model-based studies on collective punishment (e.g., [39–41]).

Compared to individual (private) punishment, IR may have some advantages as a collective punishment system. While there is little doubt that individual punishment meted out to free riders can sustain otherwise fragile cooperation, the provision of punishment suffers from a “second-order” free riding problem because non-punishers can free ride on benefits from costly punishment provided by others. IR does not suffer from this problem, as losing a future benefit (as punishment) occurs systematically, simply because an individual punisher does not exist. In addition, as punishment is not conducted individually, there is no risk of counter-punishment (punishment against the punisher), which can lower social efficiency (e.g., [42, 43]).

On the contrary, compared with private punishment, we can remark about the possible disadvantages of IR. First, in IR, while a reduction in the target harms collective profit, private punishment does not. Specifically, if an antisocial player (defector) continues to be uncooperative, IR cannot achieve the Pareto optimal situation: IR is vulnerable to antisocial behavior. The vulnerability may be attenuated by modifying IR, whereby the maximum contribution to the public good in the next period increases only if all participants contribute to the current upper bound. By contrast, we propose a modified IR, where the upper bound in the next period will rise if a certain percentage of players, say more than 90%, meet the upper bound of the present period. However, we need further empirical investigation to examine if the above-mentioned modification can attenuate IR’s vulnerability against the antisocial player.

Second, IR, as collective punishment system, may not be as effective as private punishment meted out to force free riders to cooperate, because reducing the target not only tracks down the free rider but also affects all members.

By considering the possible advantages and disadvantages mentioned above, it is thus necessary to directly compare these two systems (IR and private punishment), to verify which is better in terms of social efficiency and level of cooperation.

## Supporting Information

S1 File Experimental Instructions. (DOCX)

S2 File Experimental Data. (CSV)

S3 File Script for Statistical Analysis by R. (DOCX)
