# Supplementary material for: Institutionalize Reciprocity to Overcome the Public Goods Provision Problem
Source: PLoS One. 2016 Jun 1;11(6):e0154321. doi: 10.1371/journal.pone.0154321 (PMC4889071; doi:10.1371/journal.pone.0154321)
Supplement: S1 File — (DOCX) [file pone.0154321.s001.docx]

*After a brief verbal introduction, participants read following instructions on the computer monitor telling them that they would take part in an experiment of decision making.*

**General Guidance**

This is an experiment about decision making. You will be paid for participating, and the amount of money you earn depends on the decisions that you and the other participants make. At the end of today’s session you will be paid privately and in cash for your decisions.

You will never be asked to reveal your identity to anyone during the course of the experiment. Your name will never be associated with any of your decisions.

At this time, you will be given 500 yens (=5~6 dollars) for coming on time. All the money that you earn after this experiment will be yours to keep.

**Earnings**

In this experiment you are in a group of size 4 (you plus 3 others) and you will be asked to make a series of choices about how to allocate a set of tokens. You and the other subjects will be randomly assigned to groups, and you *will not* be told each other’s identities. But group composition remained the same throughout the experiment.

In this experiment there are 20 periods. In each period every participant is given 100 tokens and asked how amount s/he give to a group pool. The amounts every participant may choose are 0, 10, 20, 30, 40, 50 ,60, 70, 80, 90 and 100. You lose the amount you give to the pool, but the 0.4 of the sum of the tokens is given to all 4 members including you. Hence, the number of tokens you pick and the sum of tokens picked by any participant, including you, will determine the payoff you receive. Each choice that you make is similar to the following example:

**-Examples of choices you will make in this experiment and earnings**

Example 1: You are in a group of size 4 (you plus 3 others). Suppose that you and the other 3 members all give 100 tokens to a pool. You will earn:

100 (initial endowment) - 100 (the tokens you gave)

+ 0.4 * 400 (the sum of tokens 4 members gave)

=160

Example 2: You are in a group of size 4 (you plus 3 others). Suppose that you and the other 3 members all give nothing. You will earn:

100 (initial endowment) - 0 (the tokens you gave)

+ 0.4 * 0 (the sum of tokens 4 members gave)

=100

Example 3: You are in a group of size 4 (you plus 3 others). Suppose that you give 20 tokens and the other members give 50,50 & 80 tokens each. You will earn:

100 (initial endowment) - 20 (the tokens you gave)

+ 0.4 * 200(the sum of tokens 4 members gave)

=160.

You will then earn money based on the number of tokens you and the other 3 members invested in this decision. Exchange rate of 10 tokens is equal to 4 yen (*around 4~5cent*). After each period, you will know your earning and the invested tokens of all 4 members with anonymous.

*After this general instruction above, while participants allocated to control condition would start the experiment after filling out confirmation test, those who were allocated to S-IR or NL-IR conditions would read an additive instruction to understand each condition before confirmation test.*

**Additive instruction for participants in S-IR**

Please make sure that you go through following information very carefully.

- At each period, “target contribution in the period” will be shown on your computer screen. You CANNOT give the amount more than this target. For instance, when the tartget is 20, you can give 0, 10 or 20. Target is the same for all of group members.
- The “Target” of the first period is fixed at 10. But it will increase by 10 if and only if all the members give target in the previous period. If not, the target of the next period will be adjusted to the minimum points of the previous period.
  - - - Example 1: When at a period the target is 40 and all the members give 40, the target of the next period will go up by 10, that is, 50.
      - Example 2: When at a period the target is 30 and one member gives 20 but the other three members give 30, the next target will decrease to the minimum points of the previous period, 20.
    - When the target goes up to 100, it is its ceiling. It cannot be above 100.

**Additive instruction for participants in NL-IR**

Please make sure that you go through following information very carefully.

- At each period, “target contribution in the period” will be shown on your computer screen. You CAN give the amount more than this target. For instance, although the target is 20, you can give from 0 to 100. Target is the same for all of group members.
- The “Target” of the first period is fixed at 10. But it will increase by 10 if and only if all the members give at least as much as the target in the previous period. If not, the target of the next period will be adjusted to the minimum points of the previous period.
  - - - Example 1: When at a period the target is 40 and all the members give 40, the target of the next period will go up by 10, that is, 50.
      - Example 2: When at a period the target is 30 and one member gives 20 but the other three members give 30, the next target will decrease to the minimum points of the previous period, 20.
      - Example 3: When at a period the target is 70 and one member gives 90 and the other three members give 70, the target of the next period will go up by 10, that is, 80.

**Confirmation Test**

Before you start to make your decision, we should solve all questions on the paper. Read carefully through the provided information, have a look at the payoff table and write down the number of points on the paper. We will watch you solving the examples, check whether you get the right answers and help you in case that there is a problem or a question.

**Before the decision-making**

Good, now everybody has solved the problems. If anybody has any more questions raise your hand now. Otherwise let’s practice how to make your decision on your computer screen.


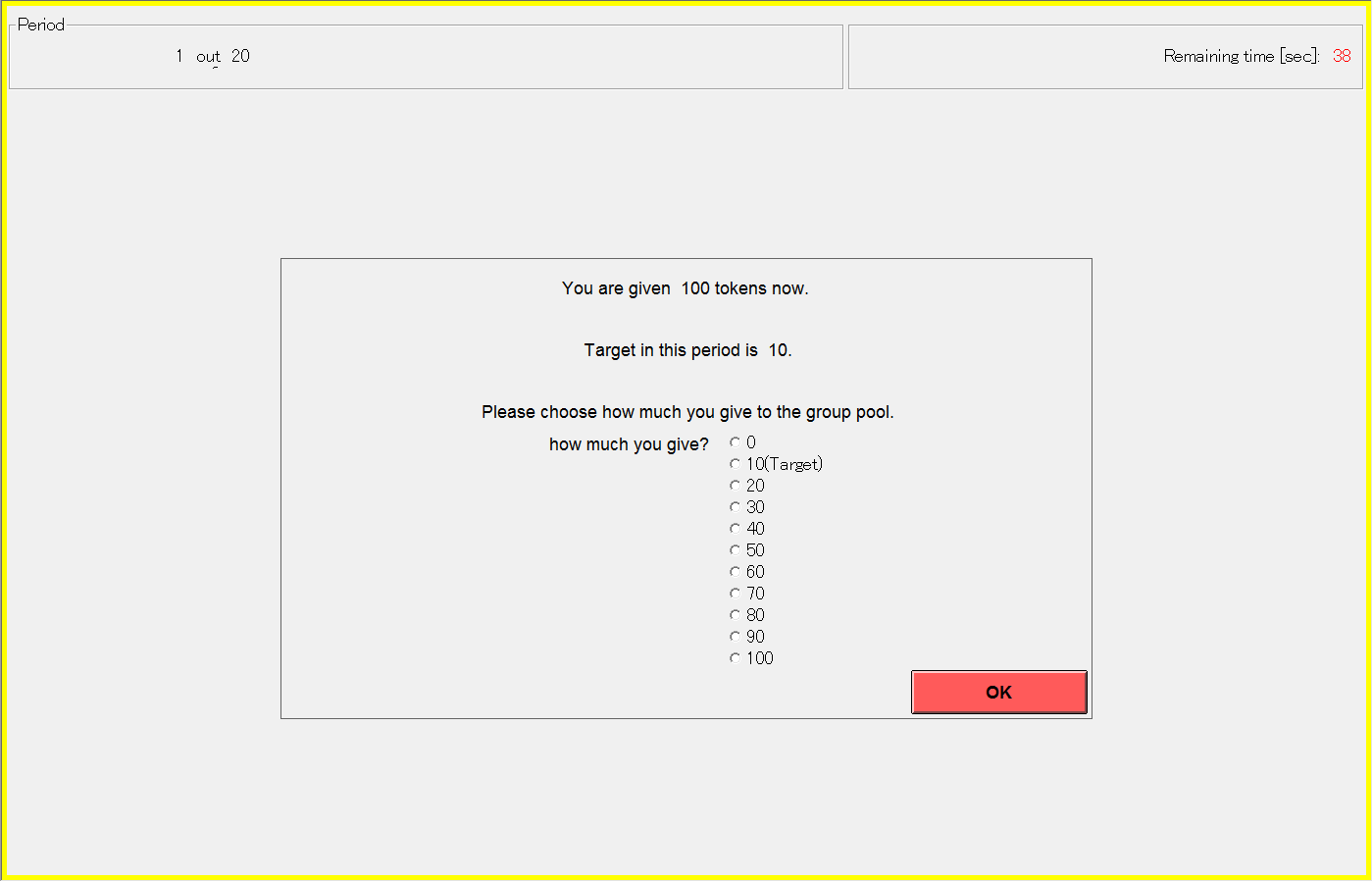


Figure 1. Screen shot of computer display when making decision　(NL-IR condition)


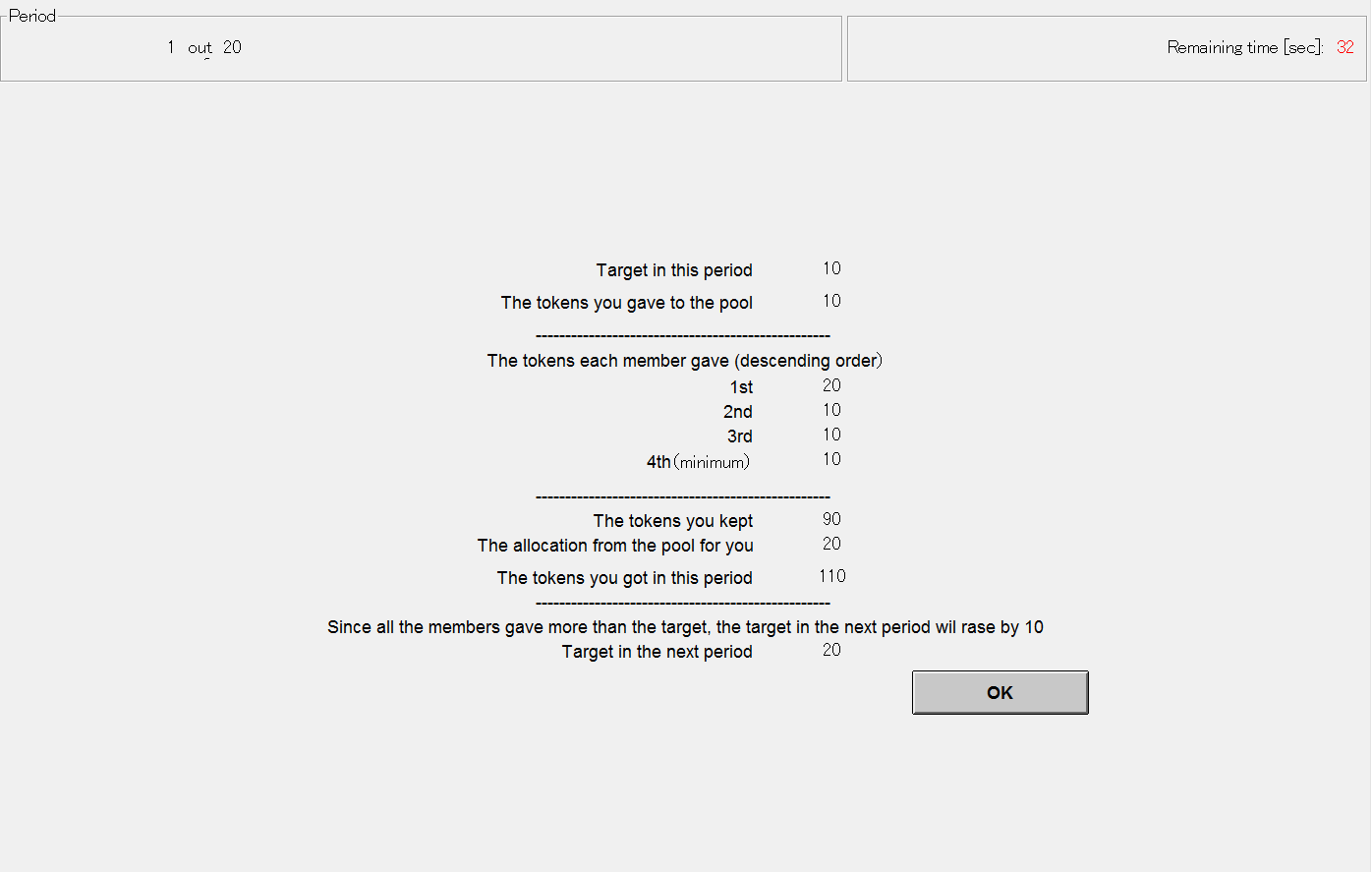


Figure 2. Screen shot of computer display when showing feedback (NL-IR condition)
