# Supplementary material for: Institutionalize Reciprocity to Overcome the Public Goods Provision Problem
Source: PLoS One. 2016 Jun 1;11(6):e0154321. doi: 10.1371/journal.pone.0154321 (PMC4889071; doi:10.1371/journal.pone.0154321)
Supplement: S3 File — (DOCX) [file pone.0154321.s003.docx]

Statistical Analysis Scripts:
*The code and data (Group_Data) in this supplement allow for the statistical analyses in the paper to be fully replicated by using R 2.12.2.*

- For results shown in Table 1

*The first part of the game (t ≤ 9)*

D_P1_9 <- subset(D, Period<=9&Group==17)

D_P1_9$Sum_Effort

mean1<-mean(D_P1_9$Sum_Effort)

D_P1_9 <- subset(D, Period<=9&Group==18)

D_P1_9$Sum_Effort

mean2<-mean(D_P1_9$Sum_Effort)

D_P1_9 <- subset(D, Period<=9&Group==19)

D_P1_9$Sum_Effort

mean3<-mean(D_P1_9$Sum_Effort)

D_P1_9 <- subset(D, Period<=9&Group==20)

D_P1_9$Sum_Effort

mean4<-mean(D_P1_9$Sum_Effort)

D_P1_9 <- subset(D, Period<=9&Group==21)

D_P1_9$Sum_Effort

mean5<-mean(D_P1_9$Sum_Effort)

D_P1_9 <- subset(D, Period<=9&Group==22)

D_P1_9$Sum_Effort

mean6<-mean(D_P1_9$Sum_Effort)

D_P1_9 <- subset(D, Period<=9&Group==23)

D_P1_9$Sum_Effort

mean7<-mean(D_P1_9$Sum_Effort)

D_P1_9 <- subset(D, Period<=9&Group==24)

D_P1_9$Sum_Effort

mean8<-mean(D_P1_9$Sum_Effort)

D_P1_9 <- subset(D, Period<=9&Group==25)

D_P1_9$Sum_Effort

mean9<-mean(D_P1_9$Sum_Effort)

D_P1_9 <- subset(D, Period<=9&Group==26)

D_P1_9$Sum_Effort

mean10<-mean(D_P1_9$Sum_Effort)

D_P1_9 <- subset(D, Period<=9&Group==27)

D_P1_9$Sum_Effort

mean11<-mean(D_P1_9$Sum_Effort)

D_P1_9 <- subset(D, Period<=9&Group==28)

D_P1_9$Sum_Effort

mean12<-mean(D_P1_9$Sum_Effort)

D_P1_9 <- subset(D, Period<=9&Group==29)

D_P1_9$Sum_Effort

mean13<-mean(D_P1_9$Sum_Effort)

D_P1_9 <- subset(D, Period<=9&Group==30)

D_P1_9$Sum_Effort

mean14<-mean(D_P1_9$Sum_Effort)

D_P1_9 <- subset(D, Period<=9&Group==31)

D_P1_9$Sum_Effort

mean15<-mean(D_P1_9$Sum_Effort)

J1_9<-c(mean1,mean2,mean3,mean4,mean5,mean6,mean7,mean8,mean9,mean10,

mean11,mean12,mean13,mean14,mean15)

D_P1_9 <- subset(D, Period<=9&Group==32)

D_P1_9$Sum_Effort

mean1<-mean(D_P1_9$Sum_Effort)

D_P1_9 <- subset(D, Period<=9&Group==33)

D_P1_9$Sum_Effort

mean2<-mean(D_P1_9$Sum_Effort)

D_P1_9 <- subset(D, Period<=9&Group==34)

D_P1_9$Sum_Effort

mean3<-mean(D_P1_9$Sum_Effort)

D_P1_9 <- subset(D, Period<=9&Group==35)

D_P1_9$Sum_Effort

mean4<-mean(D_P1_9$Sum_Effort)

D_P1_9 <- subset(D, Period<=9&Group==36)

D_P1_9$Sum_Effort

mean5<-mean(D_P1_9$Sum_Effort)

D_P1_9 <- subset(D, Period<=9&Group==37)

D_P1_9$Sum_Effort

mean6<-mean(D_P1_9$Sum_Effort)

D_P1_9 <- subset(D, Period<=9&Group==38)

D_P1_9$Sum_Effort

mean7<-mean(D_P1_9$Sum_Effort)

D_P1_9 <- subset(D, Period<=9&Group==39)

D_P1_9$Sum_Effort

mean8<-mean(D_P1_9$Sum_Effort)

D_P1_9 <- subset(D, Period<=9&Group==40)

D_P1_9$Sum_Effort

mean9<-mean(D_P1_9$Sum_Effort)

D_P1_9 <- subset(D, Period<=9&Group==41)

D_P1_9$Sum_Effort

mean10<-mean(D_P1_9$Sum_Effort)

D_P1_9 <- subset(D, Period<=9&Group==42)

D_P1_9$Sum_Effort

mean11<-mean(D_P1_9$Sum_Effort)

C1_9<-c(mean1,mean2,mean3,mean4,mean5,mean6,mean7,mean8,mean9,mean10,

mean11)

D_P1_9 <- subset(D,Period<=9&Group==1)

D_P1_9$Sum_Effort

mean1<-mean(D_P1_9$Sum_Effort)

D_P1_9 <- subset(D,Period<=9&Group==2)

D_P1_9$Sum_Effort

mean2<-mean(D_P1_9$Sum_Effort)

D_P1_9 <- subset(D,Period<=9&Group==3)

D_P1_9$Sum_Effort

mean3<-mean(D_P1_9$Sum_Effort)

D_P1_9 <- subset(D,Period<=9&Group==4)

D_P1_9$Sum_Effort

mean4<-mean(D_P1_9$Sum_Effort)

D_P1_9 <- subset(D,Period<=9&Group==5)

D_P1_9$Sum_Effort

mean5<-mean(D_P1_9$Sum_Effort)

D_P1_9 <- subset(D,Period<=9&Group==6)

D_P1_9$Sum_Effort

mean6<-mean(D_P1_9$Sum_Effort)

D_P1_9 <- subset(D,Period<=9&Group==7)

D_P1_9$Sum_Effort

mean7<-mean(D_P1_9$Sum_Effort)

D_P1_9 <- subset(D,Period<=9&Group==8)

D_P1_9$Sum_Effort

mean8<-mean(D_P1_9$Sum_Effort)

D_P1_9 <- subset(D,Period<=9&Group==9)

D_P1_9$Sum_Effort

mean9<-mean(D_P1_9$Sum_Effort)

D_P1_9 <- subset(D,Period<=9&Group==10)

D_P1_9$Sum_Effort

mean10<-mean(D_P1_9$Sum_Effort)

D_P1_9 <- subset(D,Period<=9&Group==11)

D_P1_9$Sum_Effort

mean11<-mean(D_P1_9$Sum_Effort)

D_P1_9 <- subset(D,Period<=9&Group==12)

D_P1_9$Sum_Effort

mean12<-mean(D_P1_9$Sum_Effort)

D_P1_9 <- subset(D,Period<=9&Group==13)

D_P1_9$Sum_Effort

mean13<-mean(D_P1_9$Sum_Effort)

D_P1_9 <- subset(D,Period<=9&Group==14)

D_P1_9$Sum_Effort

mean14<-mean(D_P1_9$Sum_Effort)

D_P1_9 <- subset(D,Period<=9&Group==15)

D_P1_9$Sum_Effort

mean15<-mean(D_P1_9$Sum_Effort)

D_P1_9 <- subset(D,Period<=9&Group==16)

D_P1_9$Sum_Effort

mean16<-mean(D_P1_9$Sum_Effort)

M1_9<-c(mean1,mean2,mean3,mean4,mean5,mean6,mean7,mean8,mean9,mean10,

mean11, mean12,mean13,mean14,mean15,mean16)

mean(C1_9)

mean(M1_9)

mean(J1_9)

wilcox.exact(C1_9,J1_9,paired=F)

wilcox.exact(C1_9,M1_9,paired=F)

wilcox.exact(M1_9,J1_9,paired=F)

*The second part of the game (10 ≤ t ≤ 19)*

D_P10_19 <- subset(D, Period>=10&Period<=19&Group==17)

D_P10_19$Sum_Effort

mean1<-mean(D_P10_19$Sum_Effort)

D_P10_19 <- subset(D, Period>=10&Period<=19&Group==18)

D_P10_19$Sum_Effort

mean2<-mean(D_P10_19$Sum_Effort)

D_P10_19 <- subset(D, Period>=10&Period<=19&Group==19)

D_P10_19$Sum_Effort

mean3<-mean(D_P10_19$Sum_Effort)

D_P10_19 <- subset(D, Period>=10&Period<=19&Group==20)

D_P10_19$Sum_Effort

mean4<-mean(D_P10_19$Sum_Effort)

D_P10_19 <- subset(D, Period>=10&Period<=19&Group==21)

D_P10_19$Sum_Effort

mean5<-mean(D_P10_19$Sum_Effort)

D_P10_19 <- subset(D, Period>=10&Period<=19&Group==22)

D_P10_19$Sum_Effort

mean6<-mean(D_P10_19$Sum_Effort)

D_P10_19 <- subset(D, Period>=10&Period<=19&Group==23)

D_P10_19$Sum_Effort

mean7<-mean(D_P10_19$Sum_Effort)

D_P10_19 <- subset(D, Period>=10&Period<=19&Group==24)

D_P10_19$Sum_Effort

mean8<-mean(D_P10_19$Sum_Effort)

D_P10_19 <- subset(D, Period>=10&Period<=19&Group==25)

D_P10_19$Sum_Effort

mean9<-mean(D_P10_19$Sum_Effort)

mean9

D_P10_19 <- subset(D, Period>=10&Period<=19&Group==26)

D_P10_19$Sum_Effort

mean10<-mean(D_P10_19$Sum_Effort)

D_P10_19 <- subset(D, Period>=10&Period<=19&Group==27)

D_P10_19$Sum_Effort

mean11<-mean(D_P10_19$Sum_Effort)

mean11

D_P10_19 <- subset(D, Period>=10&Period<=19&Group==28)

D_P10_19$Sum_Effort

mean12<-mean(D_P10_19$Sum_Effort)

D_P10_19 <- subset(D, Period>=10&Period<=19&Group==29)

D_P10_19$Sum_Effort

mean13<-mean(D_P10_19$Sum_Effort)

D_P10_19 <- subset(D, Period>=10&Period<=19&Group==30)

D_P10_19$Sum_Effort

mean14<-mean(D_P10_19$Sum_Effort)

D_P10_19 <- subset(D, Period>=10&Period<=19&Group==31)

D_P10_19$Sum_Effort

mean15<-mean(D_P10_19$Sum_Effort)

J10_19<-c(mean1,mean2,mean3,mean4,mean5,mean6,mean7,mean8,mean9,mean10,

mean11,mean12,mean13,mean14,mean15)

D_P10_19 <- subset(D, Period>=10&Period<=19&Group==32)

D_P10_19$Sum_Effort

mean1<-mean(D_P10_19$Sum_Effort)

D_P10_19 <- subset(D, Period>=10&Period<=19&Group==33)

D_P10_19$Sum_Effort

mean2<-mean(D_P10_19$Sum_Effort)

D_P10_19 <- subset(D, Period>=10&Period<=19&Group==34)

D_P10_19$Sum_Effort

mean3<-mean(D_P10_19$Sum_Effort)

D_P10_19 <- subset(D, Period>=10&Period<=19&Group==35)

D_P10_19$Sum_Effort

mean4<-mean(D_P10_19$Sum_Effort)

D_P10_19 <- subset(D, Period>=10&Period<=19&Group==36)

D_P10_19$Sum_Effort

mean5<-mean(D_P10_19$Sum_Effort)

D_P10_19 <- subset(D, Period>=10&Period<=19&Group==37)

D_P10_19$Sum_Effort

mean6<-mean(D_P10_19$Sum_Effort)

D_P10_19 <- subset(D, Period>=10&Period<=19&Group==38)

D_P10_19$Sum_Effort

mean7<-mean(D_P10_19$Sum_Effort)

D_P10_19 <- subset(D, Period>=10&Period<=19&Group==39)

D_P10_19$Sum_Effort

mean8<-mean(D_P10_19$Sum_Effort)

D_P10_19 <- subset(D, Period>=10&Period<=19&Group==40)

D_P10_19$Sum_Effort

mean9<-mean(D_P10_19$Sum_Effort)

D_P10_19 <- subset(D, Period>=10&Period<=19&Group==41)

D_P10_19$Sum_Effort

mean10<-mean(D_P10_19$Sum_Effort)

D_P10_19 <- subset(D, Period>=10&Period<=19&Group==42)

D_P10_19$Sum_Effort

mean11<-mean(D_P10_19$Sum_Effort)

C10_19<-c(mean1,mean2,mean3,mean4,mean5,mean6,mean7,mean8,mean9,mean10,

mean11)

D_P10_19 <- subset(D, Period>=10&Period<=19&Group==1)

D_P10_19$Sum_Effort

mean1<-mean(D_P10_19$Sum_Effort)

D_P10_19 <- subset(D, Period>=10&Period<=19&Group==2)

D_P10_19$Sum_Effort

mean2<-mean(D_P10_19$Sum_Effort)

D_P10_19 <- subset(D, Period>=10&Period<=19&Group==3)

D_P10_19$Sum_Effort

mean3<-mean(D_P10_19$Sum_Effort)

D_P10_19 <- subset(D, Period>=10&Period<=19&Group==4)

D_P10_19$Sum_Effort

mean4<-mean(D_P10_19$Sum_Effort)

D_P10_19 <- subset(D, Period>=10&Period<=19&Group==5)

D_P10_19$Sum_Effort

mean5<-mean(D_P10_19$Sum_Effort)

D_P10_19 <- subset(D, Period>=10&Period<=19&Group==6)

D_P10_19$Sum_Effort

mean6<-mean(D_P10_19$Sum_Effort)

D_P10_19 <- subset(D, Period>=10&Period<=19&Group==7)

D_P10_19$Sum_Effort

mean7<-mean(D_P10_19$Sum_Effort)

D_P10_19 <- subset(D, Period>=10&Period<=19&Group==8)

D_P10_19$Sum_Effort

mean8<-mean(D_P10_19$Sum_Effort)

D_P10_19 <- subset(D, Period>=10&Period<=19&Group==9)

D_P10_19$Sum_Effort

mean9<-mean(D_P10_19$Sum_Effort)

D_P10_19 <- subset(D, Period>=10&Period<=19&Group==10)

D_P10_19$Sum_Effort

mean10<-mean(D_P10_19$Sum_Effort)

D_P10_19 <- subset(D, Period>=10&Period<=19&Group==11)

D_P10_19$Sum_Effort

mean11<-mean(D_P10_19$Sum_Effort)

D_P10_19 <- subset(D, Period>=10&Period<=19&Group==12)

D_P10_19$Sum_Effort

mean12<-mean(D_P10_19$Sum_Effort)

D_P10_19 <- subset(D, Period>=10&Period<=19&Group==13)

D_P10_19$Sum_Effort

mean13<-mean(D_P10_19$Sum_Effort)

D_P10_19 <- subset(D, Period>=10&Period<=19&Group==14)

D_P10_19$Sum_Effort

mean14<-mean(D_P10_19$Sum_Effort)

D_P10_19 <- subset(D, Period>=10&Period<=19&Group==15)

D_P10_19$Sum_Effort

mean15<-mean(D_P10_19$Sum_Effort)

D_P10_19 <- subset(D, Period>=10&Period<=19&Group==16)

D_P10_19$Sum_Effort

mean16<-mean(D_P10_19$Sum_Effort)

M10_19<-c(mean1,mean2,mean3,mean4,mean5,mean6,mean7,mean8,mean9,mean10,

mean11)

wilcox.exact(C10_19,J10_19,paired=F)

wilcox.exact(C10_19,M10_19,paired=F)

wilcox.exact(M10_19,J10_19,paired=F)

- For results shown in Table 2

D_20M <- subset(D, Period ==20& Treatment =="M")

D_20M$Ave_Total_Profit

D_20J <- subset(D, Period ==20& Treatment =="J")

D_20J$Ave_Total_Profit

D_20C <- subset(D, Period ==20& Treatment =="C")

D_20C$Ave_Total_Profit

mean(D_20C$Ave_Total_Profit)

mean(D_20M$Ave_Total_Profit)

mean(D_20J$Ave_Total_Profit)

wilcox.exact(D_20J$Ave_Total_Profit,D_20M$Ave_Total_Profit,paired=F)

wilcox.exact(D_20J$Ave_Total_Profit,D_20C$Ave_Total_Profit,paired=F)

wilcox.exact(D_20C$Ave_Total_Profit,D_20M$Ave_Total_Profit,paired=F)
